# The State of the Art in Minimally Invasive Spine Surgery

**DOI:** 10.1155/2017/6194016

**Published:** 2017-02-28

**Authors:** Tsung-Jen Huang, Ki-Tack Kim, Hiroaki Nakamura, Anthony T. Yeung, Jiancheng Zeng

**Affiliations:** ^1^Department of Orthopedic Surgery, Taipei Medical University Hospital, Taipei, Taiwan; ^2^Department of Orthopedics, School of Medicine, College of Medicine, Taipei Medical University, Taipei, Taiwan; ^3^Department of Orthopedic Surgery, Kyung Hee University Hospital at Gangdong, Seoul, Republic of Korea; ^4^Department of Orthopedic Surgery, Osaka City University Graduate School of Medicine, Osaka, Japan; ^5^University of New Mexico School of Medicine, Albuquerque, NM, USA; ^6^Desert Institute for Spine Care, Phoenix, AZ, USA; ^7^Department of Orthopedics, West China Hospital of Sichuan University, Chengdu, China

In the past two decades, minimally invasive spine (MIS) surgery has been increasingly applied and drawn much attention in the treatment of spinal disorders [1–12]. To date, there has been a higher demand in patients' request to conduct this surgery, and the traditional open spine surgery has gradually been replaced with MIS surgery. According to the reports, the number of MIS instrumented surgeries conducted in 2010 accounted for 1/6 of the total number of all spine surgeries in the United States and 1/3 in 2016, which is anticipated to be more than 1/2 in 2020 [13].

With the aids of modern diagnostic and navigation technology, innovative spinal devices, and optical and improved MIS instruments, MIS surgery does show its merits including a smaller skin incision, less trauma to paravertebral soft tissues, reduced blood loss during operation, and a faster functional recovery in these patients. However, at present, whether MIS surgery can really achieve the expected results as in open surgery with fewer comorbidities is still debatable. However, the merits and demerits of these techniques in treating patients with spinal diseases have been systemically reviewed and critically analyzed [13–17]. The detailed information on why these techniques have low tissue invasiveness to the patient's body [18–21] and the same or even better outcome compared to traditional open spine surgery is still very limitedly elucidated. However, we are glad to see that these changes might lead to better patient surgical outcomes and reduce the economic burden [22] for the medical cost related to postoperative hospital stay or complications.

Over the past 10 years, the important role of percutaneous full endoscopic interlaminar/transforaminal surgery has been reassessed in patients with degenerative lumbar disc diseases or stenosis [23–27]. This technique has been proven to work satisfactorily as other procedures even in patients with complex spinal degeneration or mild to moderate deformity that is usually considered a reason for fusion surgery in most of our past surgeries. Furthermore, the full endoscopic interlaminar/transforaminal surgery has become a daily surgical practice in many spine centers around the world. We have seen the potentiality in these procedures which could be like the laparoscopic cholecystectomy in general surgery developed in 1987, which now has already replaced traditional open cholecystectomy. In this way, we can preserve the fusion as a fallback procedure rather than prematurely fusing the spine and we can provide our patients first with an option of nonfusion surgery.

In this special issue, 12 papers were accepted for publication after a carefully blinded review by experts in MIS or spine field.

C.-Y. Lee et al. reported a register-based case-control study of 187 patients undergoing video-assisted thoracoscopic (VATS, 111) or minimal access spine surgery (MASS, 76) in a single center. A systemic review of the literature including 625 VATS and 399 MASS patients was analyzed. The authors highlighted the notion that MASS is associated with reduced operative time, approach-related complications, and the thoracotomy conversion rate.

W. Kong et al. demonstrated nicely the surgical strategy of percutaneous full endoscopic interlaminar or extraforaminal, not transforaminal, approach for 62 patients with lumbar disc herniation. Two patients were converted to open surgery at initial procedures, with at least 1-year follow-up. The good to excellent rate of surgical result was 91.6%. The authors claimed that, based on the main location of the herniated disc and its relationship with the compressed root, percutaneous full endoscopic discectomy through 3 different puncture techniques is feasible and safe to remove the herniated disc.

M.-H. Chen and J.-Y. Chen reported on novel nonpedicular screw-based fixation in 39 patients with grade 1 lumbar spondylolisthesis with a mean follow-up of 1 year. The authors had used an interspinous fusion device (IFD) and two PLIF cages for each patient. There were no major complications noted. Interestingly, in the series, there were no spinous process fractures or migration of the IFD, however, in 5 patients having early retropulsion of the PLIF cages at the earlier weeks after surgery. They advocated that further study is mandatory for proposing a novel anatomic and radiological scoring system to identify which patients are suitable for this treatment modality and avoid postoperative complications.

P. D. Nunley et al. reported on an expanding treatment option, the Superion® spacer, an FDA approved device, for lumbar spinal stenosis at 2-, 3-, and 4-year follow-up. Certainly, this is a minimally invasive implantation procedure employing this stand-alone interspinous spacer that functions as an extension blocker to avoid compression of nerve root without direct surgical excision of tissue adjacent to it. They concluded that no inferiority was found compared with the open laminectomy group at each time period of follow-up.

M.-H. Wu et al. reported the outcome of using of the intraoperative computed tomography- (iCT-) guided navigation to operate on eight patients suffering from infectious spondylitis with simultaneous minimally invasive anterior and posterior approach. In their patients, the follow-up period was at least 2 years. They demonstrated that the application of iCT-guided navigation can provide good intraoperative 3D orientation and visualization of anatomic structures. It also offers a high pedicle screw placement accuracy in the patient's lateral decubitus position. In addition, the fact that all operation room staffs were free from the radiation exposure during operation under the iCT-guided navigation was a great advantage.

C.-L. Tai et al. performed a nicely designed research to analyze the applicability of bone cement for percutaneous vertebroplasty. The authors modified bone cement by combining polymethylmethacrylate (PMMA) with three different volume fractions of castor oil (5%, 10%, and 15%). It was found that increasing castor oil content and precooling treatment effectively decreased the peak polymerization temperatures and increased the period to reach the peak polymerization temperature. They concluded that the addition of castor oil to PMMA followed by precooling may create ideal modified bone cement with a low modulus, low polymerization temperature, and long handling time, therefore enhancing its applicability and safety for vertebroplasty.

A.-M. Wu et al. performed a systemic review and meta-analysis to investigate the outcomes of minimally invasive versus open posterior approach spinal fusion in the treatment of lumbar spondylolisthesis. They concluded that the minimally invasive posterior approach had less estimated blood loss and hospital stay than open fusion; however, the minimally invasive approach required more operative time. They also highlighted the notion that both approaches had similar results in pain and functional outcomes, complication, fusion rate, and secondary surgery.

P. H. Chou et al. made a systemic review on the “topping-off” technique by applying the hybrid stabilization device (HSD), or interspinous process device (IPD), aiming to avoid adjacent segment disease (ASD) proximal to the fusion construct. Based on their review, the incidences of radiographic ASD at index level were 12.6%, 10.2%, and 52.6% in HSD, IPD, and fusion alone, respectively. They also claimed that the application of “topping-off” technique with HSD or IPD above fusion to avoid ASD still has no good evidence. Therefore, prospective randomized clinical trials should be conducted to further elucidate the role of topping-off techniques.

W.-S. Choi et al. reported and was the first to use an endoscopic radiofrequency ablation of the sacroiliac joint complex to treat 17 patients with chronic low back pain. The clinical result was a satisfactory rate of 88.6%. With a small incision at lower posterior sacral skin after C-arm localization, then introducing the endoscope upwardly can see and ablate the branches of posterior sacral nerve effectively. Their preliminary results confirmed the feasibility and efficacy of this novel technique.

L. Kuang et al. reported a new miniopen anterolateral lumbar interbody fusion (ALLIF) with self-anchored stand-alone polyetheretherketone (PEEK) cage in 22 patients receiving lumbar revision surgery. The mean blood loss was 85.4 mL. All patients achieved solid fusion at a mean of 2-year follow-up. They found that 4 patients with 4 operated levels had cage subsidence without clinical symptoms. Significant differences were observed between the pre- and postoperation status for the VAS and ODI scores, foraminal height, and disc height. The authors advocated that this approach can lessen access-related trauma and provide good clinical results.

J. Akhgar et al. had performed an excellent investigation on the location of the common iliac veins (CIVs), with 1 mm CT-myelography slices of 504 patients, at the level of the promontorium and together with a meticulous dissection in 20 human cadavers. The authors advocated that the transarticular sacral screw trajectory is safe as long as the screw does not penetrate the anterior cortex of S1. The level of the inferior vena cava formation can help to predict the distance between the right and left CIVs at the level of the promontorium. The CIVs do not have a uniform anatomical location; therefore, preoperative computed tomography is necessary to confirm their location.

Z. Li et al. reported a series of 38 patients, 31 ipsilateral and 7 contralateral, with a recurrent lumbar disc herniation at the primary discectomy level. All the patients were treated with unilateral pedicle screws and transforaminal lumbar interbody fusion cage. The patients were followed up for a mean of 52.2 months, regardless of the laterality of the recurrence of herniation, and the authors had found no differences in clinical parameters between the two groups at follow-up except for the length of operating time. They concluded that miniopen TLIF with unilateral pedicle screw fixation can be an alternate option for single level reherniation regardless of ipsilateral or contralateral reherniation.

Thus far, we may say that MIS surgery is still in its evolving stage. Issues such as the learning curves, the need of training in anterior spine surgery when conversion to open surgery is necessary, costs and benefits, and potential complications still require constant analyses. Moreover, radiation exposure continues to be a major concern to the staffs in the operation room in MIS surgery. We hope the readers could get some inspirations from the published articles in this special issue and continue to improve our spine services.

*Tsung-Jen Huang* *Ki-Tack Kim* *Hiroaki Nakamura* *Anthony T. Yeung* *Jiancheng Zeng*
